# Supplementary material for: WASH maintains NKp46+ ILC3 cells by promoting AHR expression
Source: Nat Commun. 2017 Jun 7;8:15685. doi: 10.1038/ncomms15685 (PMC5467242; doi:10.1038/ncomms15685)
Supplement: Supplementary Information — Supplementary Figures [file ncomms15685-s1.pdf]

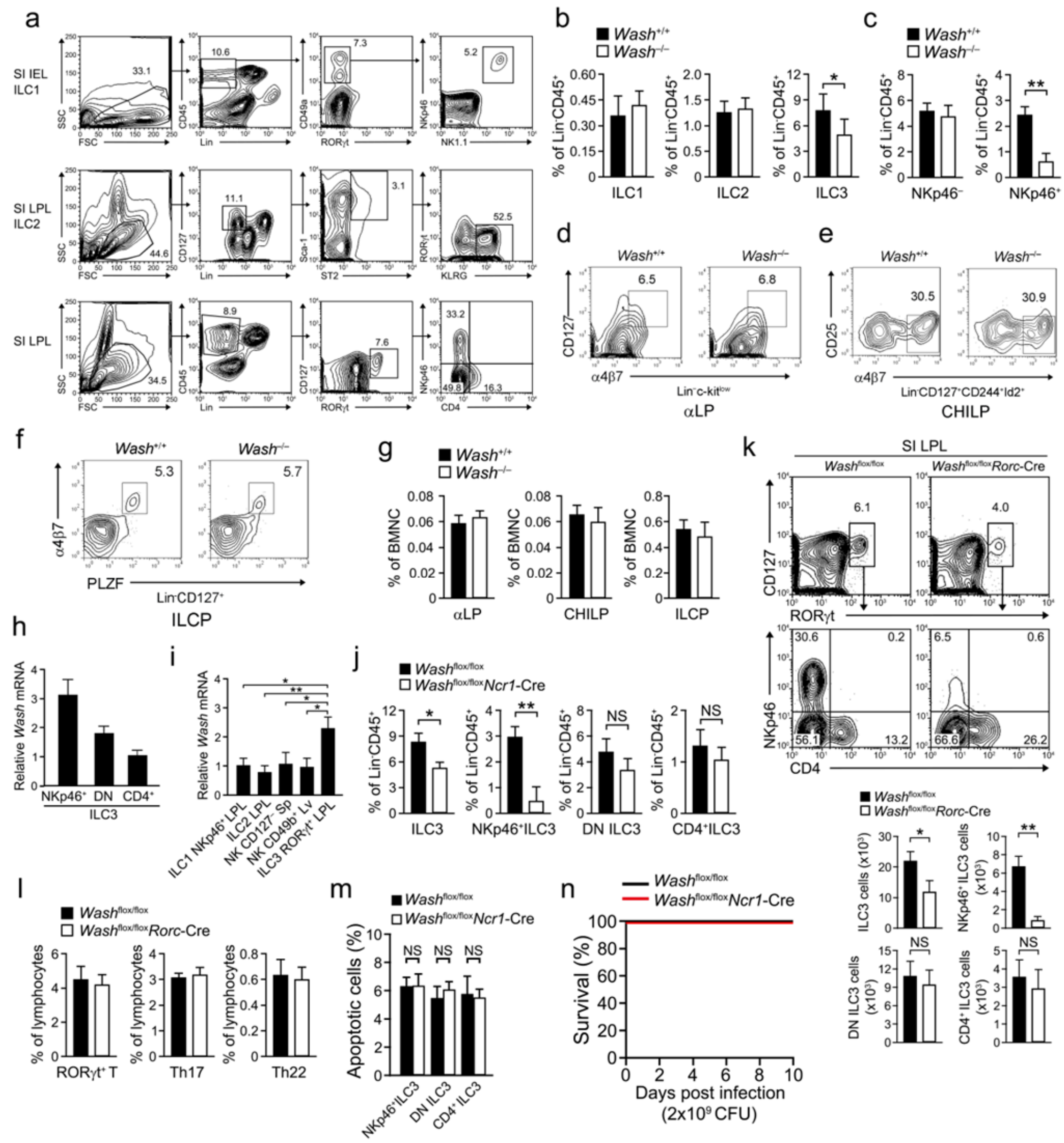

Supplementary Figure 1. WASH deficiency decreases cell numbers of NKp46^{+} ILC3s. (a) Gating strategies for gating out ILC1, ILC2 and ILC3 cells from small intestines. ILC1 cells were gated out from Lin^{-}CD45^{+}RORγt^{-}CD49a^{+}NK1.1^{+}NKp46^{+} SI IEL cells. ILC2 cells were gated out from Lin^{-}CD127^{+}ST2^{+}Sca-1^{+}KLRG^{+}RORγt^{-} SI LPL cells and ILC3 were gated out from Lin^{-}CD45^{+}CD127^{+}RORγt^{+} SI LPL cells. (b) ILCs from *Wash*$^{flox/flox}$ and *Wash*$^{flox/flox}$*Mx1*-Cre mice 3 weeks post poly(I:C) administration were calculated by flow cytometry. Percentages of ILCs in small intestines of *Wash*$^{flox/flox}$ (*Wash*$^{+/+}$) and *Wash*$^{flox/flox}$*Mx1*-Cre (*Wash*$^{-/-}$) mice were calculated. Sorting strategies: Lin^{-}CD45^{+}RORγt^{-}CD49a^{+}NK1.1^{+}NKp46^{+} for ILC1, Lin^{-}CD127^{+}ST2^{+} Sca-1^{+}KLRG^{+} RORγt^{-} for ILC2, and Lin^{-}CD45^{+}CD127^{+}RORγt^{+} for ILC3. (c) Percentages of NKp46^{-} and NKp46^{+} ILC3s in small intestines of the indicated mice were calculated. (d-f) Gating strategies for analyzing ILC progenitors in bone marrow. Sorting makers: Lin^{-} cKit^{low} CD127^{+}α4β7^{+} for αLP, Lin^{-}CD127^{+}α4β7^{+} PLZF^{+} for ILCP, and Lin^{-} CD127^{+}α4β7^{+} CD25^{-}CD244^{+}Id2^{+} for CHILP. (g) Percentages of ILC progenitor cells in BM of the indicated mice. Sorting makers: Lin^{-} cKit^{low} CD127^{+}α4β7^{+} for αLP, Lin^{-}CD127^{+}α4β7^{+} PLZF^{+} for ILCP, and Lin^{-}

$CD127^+\alpha4\beta7^+$ $Flt3^-$ $CD25^-CD27^+Id2^+$ for CHILP. (h, i) WASH mRNA levels in the indicated cell subsets were examined through RT-PCR. (j) SI LPLs of *Wash*$^{flox/flox}$ and *Wash*$^{flox/flox}$*Ncr1*-Cre mice were examined through flow cytometry. Cells were gated out from $CD45^+CD19^-CD3^-$ cells. Percentages of ILC3s and indicated subpopulations of ILC3s were calculated. (k) Flow cytometry analysis of lamina propria lymphocytes of small intestine (SI ILP) of *Wash*$^{flox/flox}$ and *Wash*$^{flox/flox}$*Rorc*-Cre mice. Cells were gated out from $CD45^+CD19^-CD3^-$ cells (upper panel). Numbers of ILC3s and indicated subpopulations of ILC3s were calculated (lower panel). n=6. (l) Percentages of the indicated cells were calculated in SI LPLs by flow cytometry in *Wash*$^{flox/flox}$ and *Wash*$^{flox/flox}$*Rorc*-Cre mice. Sorting markers: $CD4^+ROR\gamma t^+$ for $ROR\gamma t^+$ T cells, $CD4^+$ $ROR\gamma t^+IL17^+$ for Th17 cells, and $CD4^+$ $ROR\gamma t^+IL22^+$ for Th22 cells. (m) The indicated live cells were sorted from SI LPLs of *Wash*$^{flox/flox}$;RORγt-GFP or *Wash*$^{flox/flox}$*Ncr1*-Cre;RORγt-GFP mice, followed by Annexin V and PI staining. Cells positive for Annexin V and PI were counted as apoptotic cells. n=5. (n) 8 wk-old *Wash*$^{flox/flox}$ and *Wash*$^{flox/flox}$*Ncr1*-Cre mice were orally infected with *C. rodentium* ($2x10^9$ CFU per mouse), followed by survival rate analysis. n=10. Data are shown as means±SD. $*$, $P<0.05$; $**$, $P<0.01$; $***$, $P<0.001$. Data are representative of at least three independent experiments.

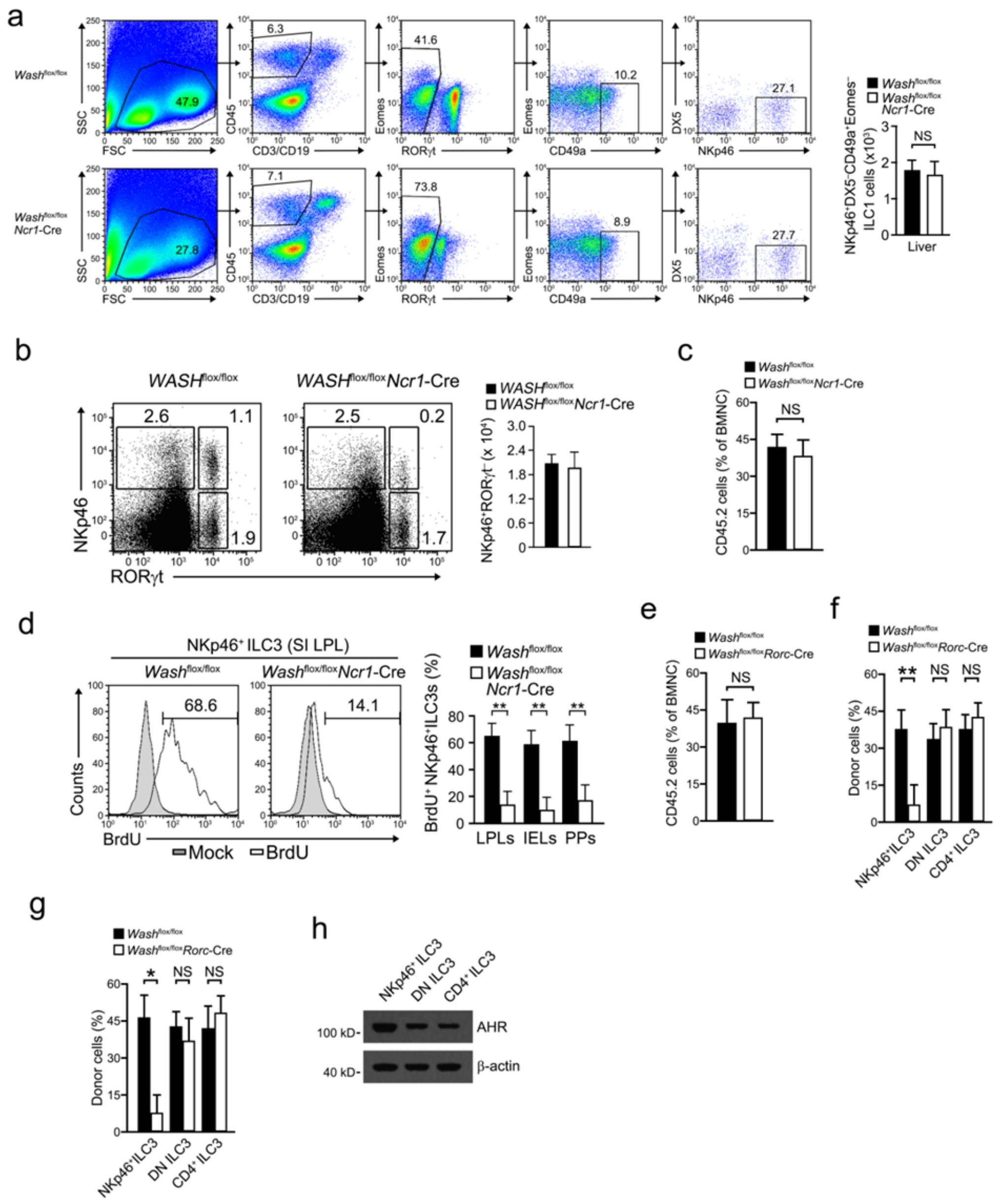

Supplementary Figure 2. WASH maintains the number of NKp46$^+$ ILC3 population intrinsically. (a) Flow cytometry analysis of liver cells from *Wash*$^{flox/flox}$ and *Wash*$^{flox/flox}$*Ncr1*-Cre mice with indicated markers (left panel). Numbers of NKp46$^+$DX5$^-$ CD49a$^+$Eomes$^-$ ILC1 cells were calculated (right panel). (b) Flow cytometry analysis of SI LPL of *Wash*$^{flox/flox}$ and *Wash*$^{flox/flox}$*Ncr1*-Cre mice. Cells were gated out from Lin$^-$CD45$^+$ cells (left panel). Numbers of NKp46$^+$RORγt$^-$ cells were calculated (right panel). (c) 2x10^6 *Wash*$^{flox/flox}$RORγt-GFP or *Wash*$^{flox/flox}$*Ncr1*-CreRORγt-GFP BM cells were co-transplanted with 2x10^6 CD45.1 BM cells into lethally irradiated CD45.1 recipient mice, followed by donor chimerism examination of bone marrow nucleated cells (BMNC) 8 wk later. (d) Mice reconstituted with *Wash*$^{flox/flox}$RORγt-GFP or *Wash*$^{flox/flox}$*Ncr1*-CreRORγt-GFP BM cells were intraperitoneally injected with 7 mg/kg BrdU for 16 h, followed by flow cytometry analysis of BrdU signals in the indicated NKp46$^+$ ILC3 cells. Percentages of BrdU positive cells were calculated. n=5. (e) 2x10^6 *Wash*$^{flox/flox}$RORγt-GFP or

Wash$^{flox/flox}$*Rorc*-CreRORγt-GFP BM cells were co-transplanted with 2x10^6 CD45.1 BM cells into lethally irradiated CD45.1 recipient mice, followed by donor chimerism examination of bone marrow nucleated cells (BMNC) 8 wk later. (f, g) 2x10^6 *Wash*$^{flox/flox}$;RORγt-GFP or *Wash*$^{flox/flox}$*Rorc*-Cre; RORγt-GFP bone marrow cells were co-transplanted with 2x10^6 CD45.1 bone marrow cells into lethally irradiated CD45.1 recipient mice, followed by donor chimerism examination of the indicated ILC3 subpopulations in small intestine (f), and large intestine (g) 8 wk later through flow cytometry. Donor chimerisms were calculated. n=6. (h) AHR protein levels were examined by immunoblotting with antibody against AHR in indicated ILC3 subsets sorted from RORγt-GFP reporter mice. 1x10^5 cells were pooled from 5 mice. Experiment was repeated for two times. Data are shown as means±SD. *, $P<0.05$; **, $P<0.01$; ***, $P<0.001$. Data are representative of at least three independent experiments unless mentioned.

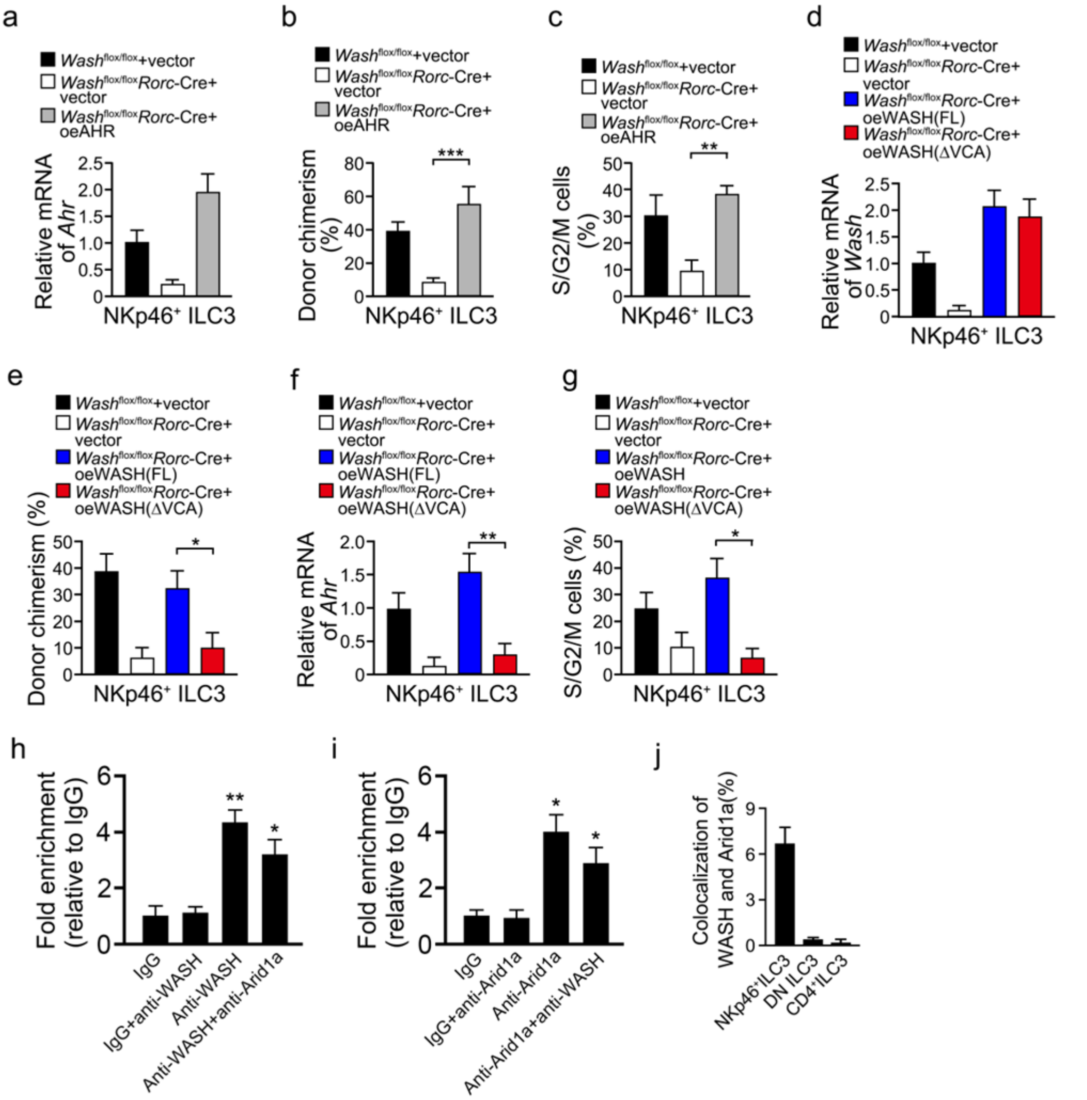

Supplementary Figure 3. WASH is required for AHR expression in NKp46^{+} ILC3s. (a-c) *Wash*$^{flox/flox}$;RORγt-GFP and *Wash*$^{flox/flox}$*Rorc*-Cre;RORγt-GFP BM cells transfected with full-length AHR (a) were co-transplanted with equal numbers of CD45.1 recipient BM cells into lethally irradiated mice, followed by donor chimerism examination (b) and cell cycle analysis (c) of NKp46^{+} ILC3s 8 wk later. For (a-c), n=7. (d-g) *Wash*$^{flox/flox}$;RORγt-GFP and *Wash*$^{flox/flox}$*Rorc*-Cre;RORγt-GFP BM cells transfected with WASH(FL) or WASH(ΔVCA) (d) were co-transplanted with equal numbers of CD45.1 recipient BM cells into lethally irradiated mice, followed by examination donor chimerism (e), AHR expression (f) and cell cycle (g) of NKp46^{+} ILC3s 8 wk later. For (d-g), n=6. (h) ChIP-ReChIP of *Ahr* promoter with antibodies against WASH or Arid1a. NKp46^{+} ILC3 cells sorted from RORγt-GFP mice were subjected to ChIP assay with IgG or anti-WASH antibody, followed by ReChIP with antibodies against WASH or Arid1a. *Ahr* promoter was detected by PCR. (i) NKp46^{+} ILC3 cells sorted from RORγt-GFP mice were subjected to ChIP assay with IgG or anti-Arid1a antibody, followed by ReChIP with antibodies against Arid1a or WASH. (j) Indicated cells were sorted from SI LPLs of WT mice, followed by immunostaining of WASH and Arid1a with antibodies against WASH and Arid1a.

Co-localization rates between WASH and Arid1a were calculated. At least 100 cells were calculated. Data are shown as means±SD. *, $P<0.05$; **, $P<0.01$; ***, $P<0.001$. Data are representative of at least three independent experiments.

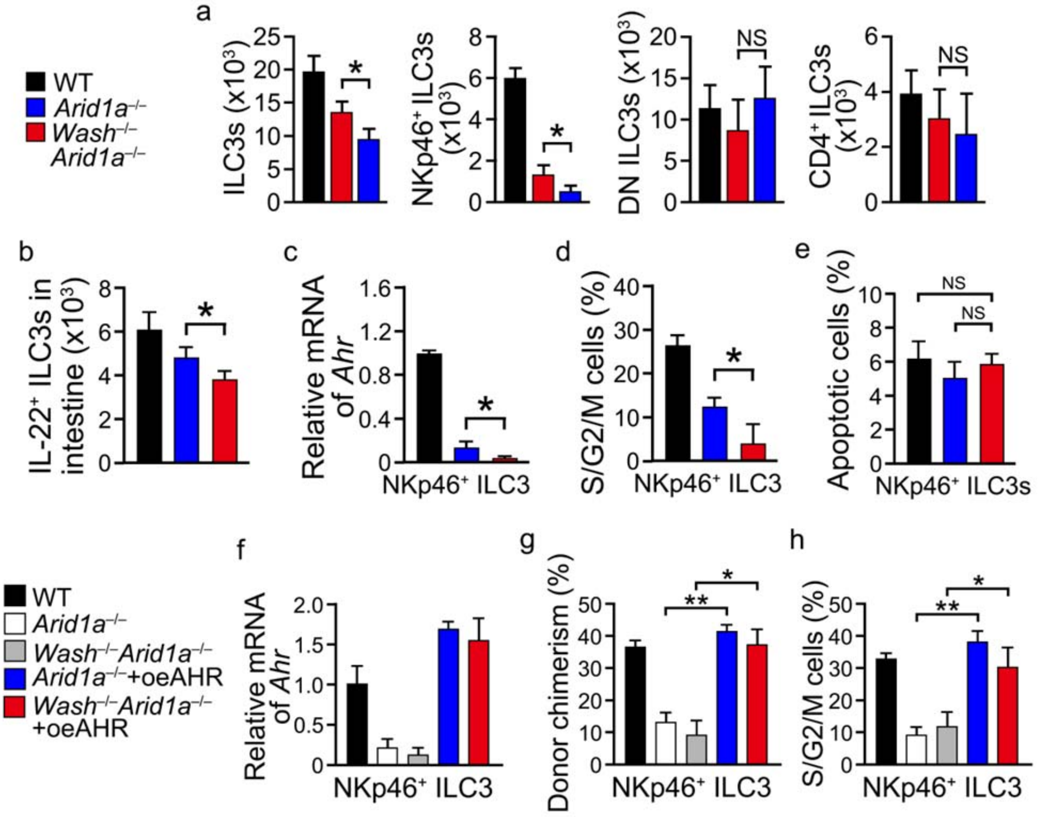

Supplementary Figure 4. WASH associates Arid1a to promote AHR expression. (a) Numbers of ILC3s and the indicated subpopulations of ILC3s were calculated in small intestines of WT, *Arid1a*$^{-/-}$ and *Wash*$^{-/-}$*Arid1a*$^{-/-}$ mice. (b) Numbers of IL-22 producing ILC3s were calculated in small intestines of WT, *Arid1a*$^{-/-}$ and *Wash*$^{-/-}$*Arid1a*$^{-/-}$ mice infected with *C. rodentium*. (c) *Ahr* mRNA expression was examined in NKp46+ ILC3s from WT, *Arid1a*$^{-/-}$ and *Wash*$^{-/-}$*Arid1a*$^{-/-}$ mice. (d) Cell cycle analysis of NKp46^{+} ILC3s from WT, *Arid1a*$^{-/-}$ and *Wash*$^{-/-}$*Arid1a*$^{-/-}$ mice. For (a-d), n=7. (e) The indicated live cells were sorted from SI LPLs of WT, *Arid1a*$^{-/-}$ and *Wash*$^{-/-}$*Arid1a*$^{-/-}$ mice carrying RORγt-GFP reporter, followed by Annexin V and PI staining. Cells positive for Annexin V and PI were counted as apoptotic cells. n=5. (f-h) WT, *Arid1a*$^{flox/flox}$*Rorc*-Cre and *Wash*$^{flox/flox}$*Arid1a*$^{flox/flox}$*Rorc*-Cre BM cells overexpressed with AHR were co-transplanted with equal numbers of CD45.1 recipient BM cells into lethally irradiated mice, followed by examination of AHR expression (f), donor chimerism (g) and cell cycle (h) of NKp46^{+} ILC3s 8 wk later. For (f-h), n=6. Data are shown as means±SD. ∗, *P*<0.05; ∗∗, *P*<0.01; ∗∗∗, *P*<0.001. Data are representative of at least three independent experiments.

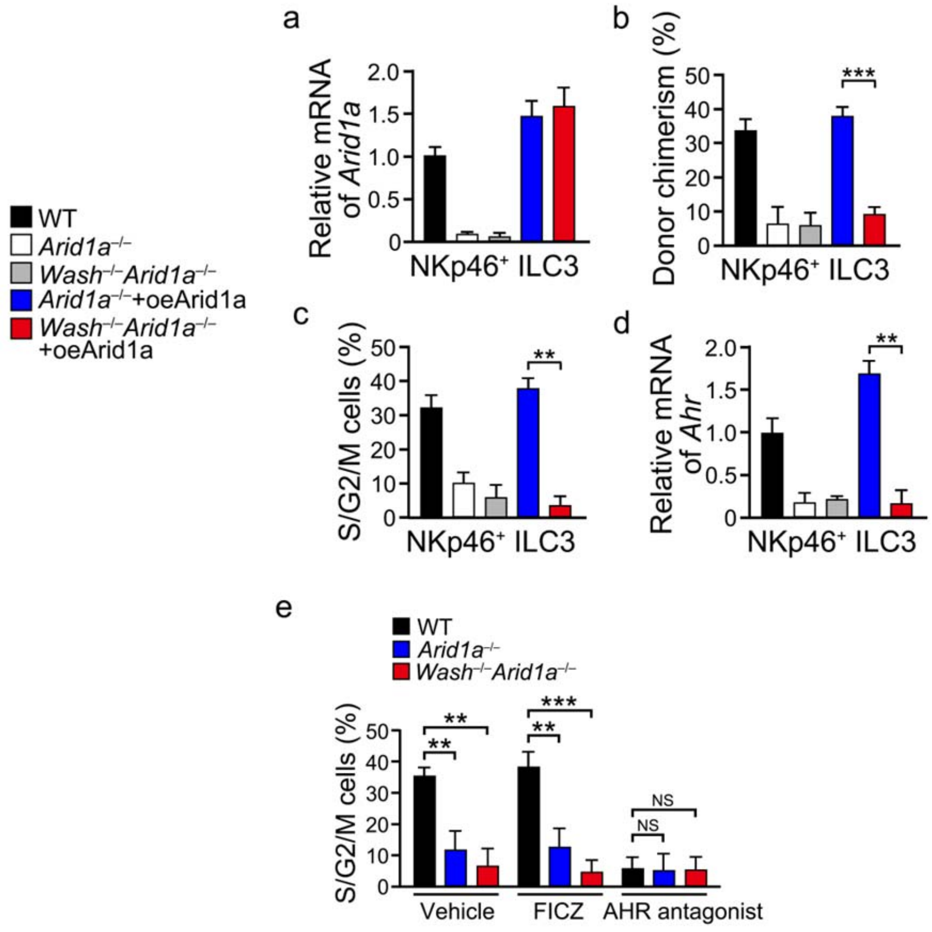

Supplementary Figure 5. Arid1a maintains the expansion of NKp46$^+$ ILC3s in a WASH-dependent manner. (a-d) RORγt-GFP expressing WT, *Arid1a*$^{flox/flox}$*Rorc*-Cre and *Wash*$^{flox/flox}$*Arid1a*$^{flox/flox}$*Rorc*-Cre BM cells were transfected with Arid1a and co-transplanted with equal numbers of CD45.1 recipient BM cells into lethally irradiated mice, followed by examination of *Arid1a* expression (a), donor chimerism (b), cell cycle (c) and AHR expression (d) of NKp46$^+$ ILC3s 8 wk later. For (a-d), n=5. (e) WT, *Arid1a*$^{-/-}$ and *Wash*$^{-/-}$*Arid1a*$^{-/-}$ NKp46$^+$ ILC3s were sorted from RORγt-GFP expressing WT, *Arid1a*$^{flox/flox}$*Rorc*-Cre and *Wash*$^{flox/flox}$*Arid1a*$^{flox/flox}$*Rorc*-Cre mice, followed by treatment with FICZ (100 nM) or AHR antagonist (10 μm). Cell cycle statuses of the indicated cells were examined 18 h later. Data are shown as means±SD. ∗, $P<0.05$; ∗∗, $P<0.01$; ∗∗∗, $P<0.001$. Data are representative of at least three independent experiments.

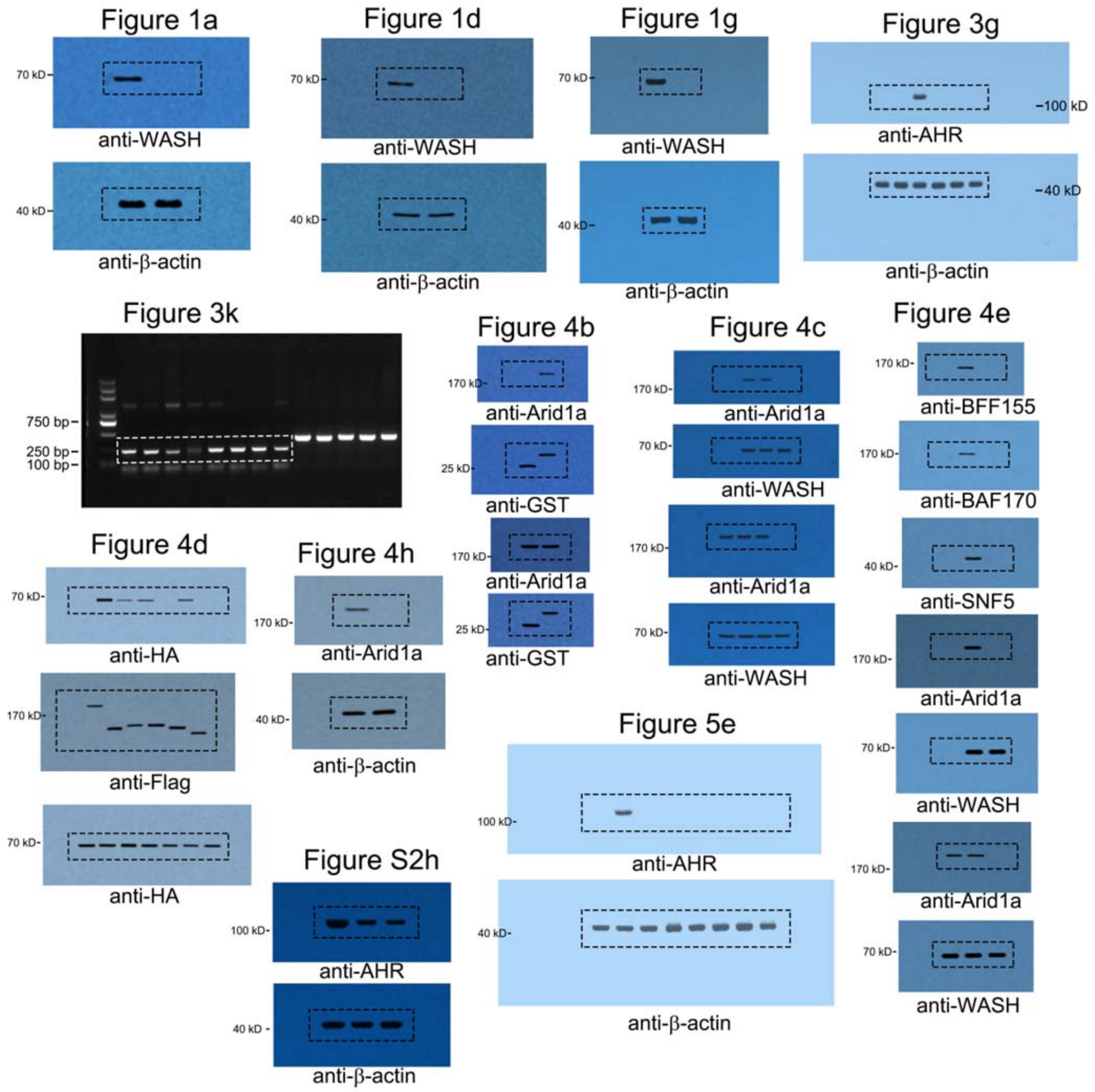

Supplementary Figure 6. Uncropped images for this study.
